# Yeast derivatives as a source of bioactive components in animal nutrition: A brief review

**DOI:** 10.3389/fvets.2022.1067383

**Published:** 2023-01-06

**Authors:** Rob Patterson, Anna Rogiewicz, Elijah G. Kiarie, Bogdan A. Slominski

**Affiliations:** ^1^CBS BioPlatforms Inc., Calgary, AB, Canada; ^2^Department of Animal Science, University of Manitoba, Winnipeg, MB, Canada; ^3^Department of Animal Biosciences, University of Guelph, Guelph, ON, Canada

**Keywords:** yeast, bioactives, livestock, health, nutrition

## Abstract

With a long history of inclusion within livestock feeding programs, yeast and their respective derivatives are well-understood from a nutritional perspective. Originally used as sources of highly digestible protein in young animal rations in order to offset the use of conventional protein sources such as soybean and fish meal, application strategies have expanded in recent years into non-nutritional uses for all animal categories. For the case of yeast derivatives, product streams coming from the downstream processing of nutritional yeast, the expansion in use cases across species groups has been driven by a greater understanding of the composition of each derivative along with deeper knowledge of mechanistic action of key functional components. From improving feed efficiency, to serving as alternatives to antibiotic growth promoters and supporting intestinal health and immunity while mitigating pathogen shedding, new use cases are driven by a recognition that yeast derivatives contain specific bioactive compounds that possess functional properties. This review will attempt to highlight key bioactive categories within industrially applicable yeast derivatives and provide context regarding identification and characterization and mechanisms of action related to efficacy within a range of experimental models.

## Introduction

Yeast are single celled, eukaryotic organisms that belong to the fungi kingdom. With size ranging from 3 to 4 μm, they possess cell walls, nuclear membranes, but unlike plant cells do not contain chloroplasts (1). Yeasts are key member of nutrient recycling as they rely on other organisms, be it alive or dead for nutrients and obtain these through the production and secretion of proteolytic, glycolytic and or lipolytic enzymes to digestion organic matter into usable nutrients (2). Reproduction occurs through budding and fission (3), whereby budding is the process of parent cell expansion into a protrusion or “bud” along the cell wall that separates from the parent or expands further into a ribbon structure of yeast buds. Fission is the process where a parent cell expands and divides into two daughter cells (4). Yeast can survive in aerobic or anaerobic environments and are therefore described as facultative anaerobes (5). However, yeasts tend to prefer aerobic conditions for propagation, as the production of carbon dioxide and energy from oxygen and sugars is more efficient than under anaerobic conditions where the end product of metabolism tends to be ethanol (6, 7).

The inclusion of yeast into livestock feeds has occurred under many use cases for many decades (8, 9). Traditionally, yeast, either whole or derivations thereof, were included into livestock feeds as protein sources due to their high digestibility and optimal ratio of essential amino acids (10). These yeast streams had many origins including from primary yeast production factories as well as being by-products from ethanol production. Inclusion into livestock rations was a logical endpoint for these co-streams as they generally had high nutritional value, were inexpensive and had good palatability profiles which would improve feed consumption. However, over time, a greater knowledge base has developed pertaining to the functional properties of intact yeast as well as yeast derivatives which has led to a non-nutritive use case for supplementing yeast into livestock rations in recent years (11, 12). Here, the focus has been on using the cell wall fraction of yeast cells (13, 14) or on using the nutrient rich cytosol fraction (15), both of which are streams that originate after the lysis of whole, live or inactivated, yeast cells. It has been estimated that the combined value of the feed yeast market will reach $3.96 billion US dollars by 2026 (https://www.reportlinker.com/p06320094/Feed-Yeast-Global-Market-Report.html?utm_source=GNW), with the majority of growth and value coming from the yeast derivative market segments. Given the interest and continued usage and adoption of these novel technologies, this paper will focus on reviewing bioactive compounds derived from yeast cell wall and yeast cytosol fractions in order to provide better context for participants in the animal nutrition industry.

## Yeast processing

**Figure 1:**
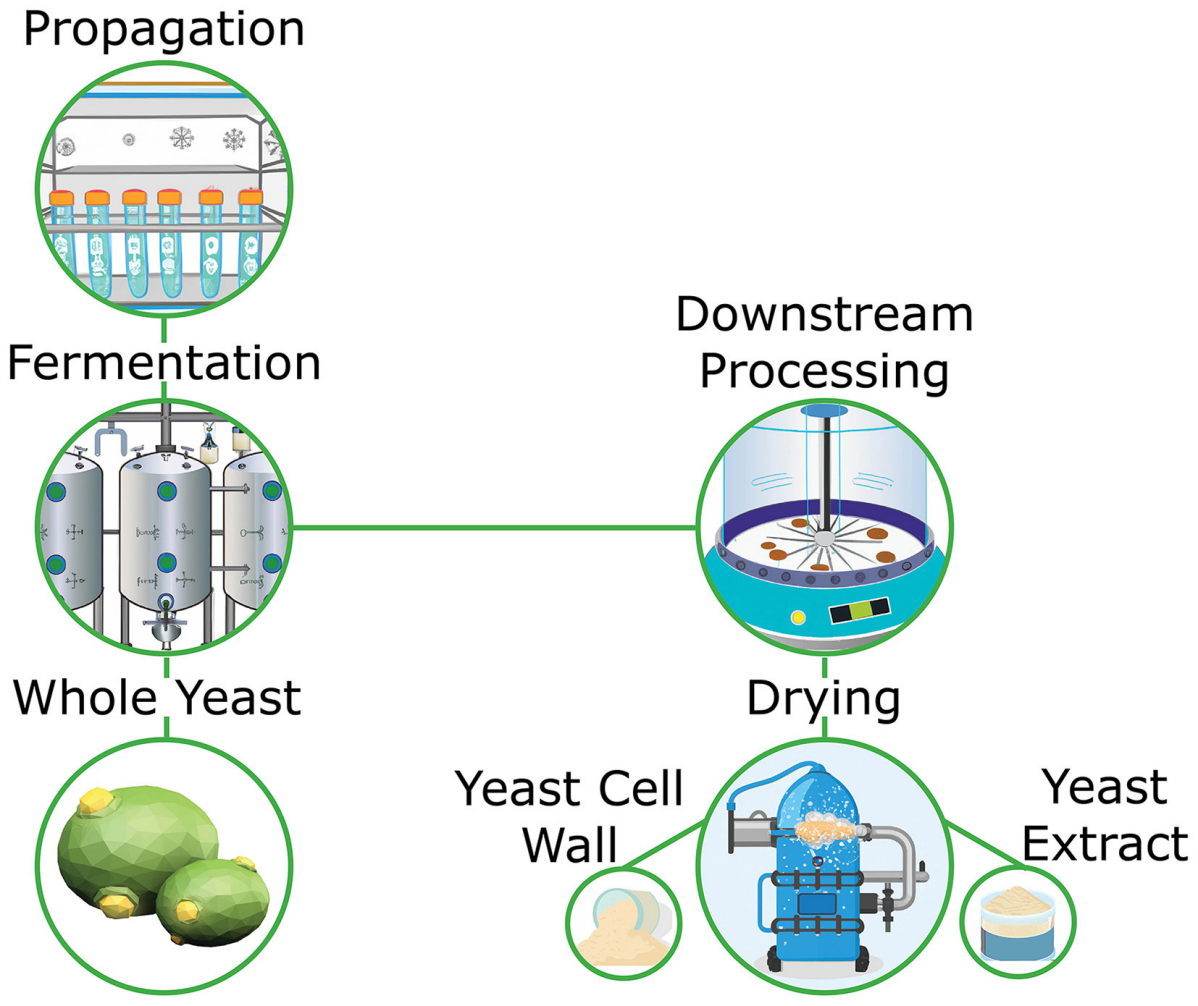
Overview of whole yeast and yeast derivative production.

Primary yeast cultures are typically proprietary strains that are developed for specific purposes. For example, select strains of *Saccharomyces cerevisiae* have been developed for the production of breads that are excellent producers of carbon dioxide, which is important in the leavening process of dough production (16). Similarly, *Pichia pastoris*, which is used in the industrial production of biotherapeutics, is grown under specific conditions in order to maximize expression of target compounds (17). These strains require propagation through a series of lab scale growth steps prior to large scale propagation (Figure 1). When considering yeast for application into the food and beverage industries the final product can take the form of cream yeast or dried yeast, depending on the application of the final user, while in pharmaceutical and research based uses, it is the expression products from a given yeast organism that is of interest.

As previously mentioned, downstream processing can be applied during the propagation steps of yeast in order to inactivate live yeast or lyse whole yeast into its constituent parts. Typically, initial processing steps involve manipulation of temperature, pH and osmotic balance in order to facilitate cell lysis (18, 19). Once cells are lysed, centrifugation can be applied to split fractions into insoluble carbohydrate rich cell wall fractions and soluble protein rich cytosolic fractions. Filtration can be used in place of or in conjunction with centrifugation in order to further partition each phase depending on the desired purity or composition of each fraction (20). Finally, water is removed thereby increasing the concentration of bioactive compounds within each fraction. Spray drying is typically used for dewatering, however, box and drum drying can also be utilized depending on the throughput and compositional integrity requirements.

## Proximal composition of intact nutritional yeast

The composition of nutritional yeast and yeast derivatives are considerably different and have been reviewed extensively (21). From a nutritional standpoint, intact nutritional yeasts such as brewers and torula, which are commonly referred to as nutritional yeasts in the context of animal nutrition, typically possess high levels of crude protein and low levels of crude fat and fiber. For context, and as was described previously, yeasts are single celled organisms which is a critical characteristic as it differentiates them from many other nutritional feedstuffs that originate from multicellular plant or animal sources. For example, plant proteins usually originate in the seed or fruit structure and as such contain not only protein but fractions of the seed hull and cotyledon which are high in fibers such as cellulose, hemi-cellulose, pectin and lignin (22). As single cell organisms, yeast have a cell wall in place of a hull and are therefore lower in fiber as compared to plant based feedstuffs. Furthermore, as yeast rely on external nutrient sources, they possess limited intracellular fat storage, which contrasts to plant based feed ingredients which store fat for germination within oleosomes (23). Thus, nutritional yeasts, be they brewers, bakers or torula tend to be high in crude protein and low in crude fat and fiber, respectively. Although conventional fiber measurements such as crude fiber, result in low values compared to plant based feedstuffs, yeast do contain substantial amounts of what could be described as dietary fiber, which are predominantly mannan glucose based non-starch polysaccharides (NSP; Table 1). Although fiber within intact yeasts is typically low for the aforementioned reasons, the variability within and between yeasts is notable. From Table 1 it can be seen that on average, mannan and glucose account for all NSP residues in bakers and brewers yeast and combine for >95% of the total NSP fraction in torula yeast. The standard deviation for mannose and glucose is >25% of the mean in bakers/brewers yeast and <8% in torula yeast, indicating a greater degree of variability in the former as compared to torula yeast. Given that bakers /brewers yeast has greater use cases and therefore has a wider breadth of production conditions as compared to torula yeast, the described differences for each intact yeast source with respect to fibrous constituents is as expected.

**Table 1:** Nutrient composition of intact nutritional yeast sources^a^.

**Nutrient, % as is**	**Mean**	**Std Dev**	**Min**	**Max**
**Bakers and brewers yeast (** ***Saccharomyces cerevisiae*** **)**
Dry matter	95.10	1.34	93.43	96.90
Crude protein (*N* x 6.25)	44.75	5.50	39.75	56.41
Starch	4.51	2.89	n.d.^b^	17.5
Ether extract	1.62	0.61	0.35	2.26
Ash	5.19	0.30	4.87	5.86
Crude fiber	1.90	1.30	0.1	4.4
Neutral detergent fiber (NDF)	10.05	9.40	n.d.	20.7
Acid detergent fiber (ADF)	5.87	2.50	n.d.	5.70
Non-starch polysaccharides	20.21	6.54	14.47	33.19
Glucan/glucose	10.38	2.90	6.46	14.34
Mannan/mannose	7.81	4.17	0.55	14.80
Lysine	4.54	0.90	4.60	7.60
Methionine	1.05	0.30	1.30	2.20
Cysteine	0.62	0.60	0.33	1.9
Threonine	3.18	0.70	3.70	5.60
Tryptophan	0.79	0.20	1.00	1.40
Calcium	0.29	0.11	0.10	0.54
Phosphorus	1.31	0.24	0.96	2.00
Nucleotides	0.06	0.08	n.d.	0.11
**Torula yeast (** ***Candida utilis*** **)**
Dry matter	94.83	0.62	94.00	95.83
Crude protein (*N* x 6.25)	52.04	0.66	51.44	53.48
Ether extract	0.16	0.10	0.07	0.36
Ash	8.44	0.60	7.04	8.88
Crude fiber	2.07	2.79	0.08	6.2
Non-starch polysaccharides	21.83	1.75	19.17	23.87
Glucan/glucose	11.82	0.76	10.60	13.32
Mannan/mannose	9.28	1.37	7.17	10.46
Arabinose	0.73	0.23	0.43	0.98
Lysine	4.88	1.36	3.45	6.68
Methionine	0.76	0.34	0.38	1.14
Cysteine	0.74	0.27	0.49	1.02
Threonine	3.26	1.10	2.35	4.55
Tryptophan	0.57	0.07	0.52	0.62
Calcium	0.23	0.09	0.13	0.47
Phosphorus	2.43	0.61	1.78	0.61
Nucleotides	1.44	1.81	0.04	3.49

a Adapted from: CBS Bio-Platforms Inc. internal analyses 2020-2022; Evonik AMINODat 6.1 2021; Feedipedia.org 2022.

b Not detected.

The protein and mineral content of nutritional yeasts is relatively consistent across groupings. Table 1 shows that bakers/brewers and torula yeast have average crude protein levels of 44.75 and 52.04%, respectively. For mineral content, as measured by ash, bakers/brewers yeast has on average 5.19% while torula has 8.44%, with both categories having relatively little variability associated with this measurement (Table 1). Crude protein variability of bakers/brewers yeast is greater than that of torula when looking at standard deviation as a percent of the mean, which as detailed previously likely relates to broader application of the former. It is worth noting that typically, crude protein is determined by calculating total nitrogen content of an ingredient and multiplying this value by 6.25, however, a coefficient of 5.8 has been reported to be used for yeasts which could be a source of variability seen in the literature (24). Amino acid profiles of each yeast category are relatively similar with lysine being 4.54% in bakers/brewers and 4.88 in torula as an example. Additionally, it should be pointed out that crude ash encompasses key minerals such as phosphorus and calcium and given the importance of these minerals it follows that total mineral content variability would be low. For example, phosphorus, the major mineral component of nutritional yeasts, is required for energy metabolism as a component of ATP (7). Furthermore, calcium, along with phosphorus, is used within numerous cell signaling pathways (25). Although propagation techniques will lead to differences in the nominal amount of each nutrient category, it is not surprising that these nutrients are quantitatively consistent due to the critical functions they are a part of within the yeast cell.

Although the intestinal benefits of yeast cell are well-recognized, as the biotechnology progresses, more attention has been paid to value-added yeast-derived products (26, 27). The bioactivity of yeast cell components can be substantially enhanced by enzymatic modification of the cell wall polysaccharides, or by releasing and separating bioactive fractions, such as nucleic acids (28). The enzymatic hydrolysis of long-chain polysaccharides of the cell wall will result in shortening of the chain and production of more potent water-soluble derivatives, which can therefore be more effective in the gut environment than the intact yeast (29). The extracted intracellular components of yeast might exert more pronounced bioactivities, mainly due to their superior bioavailability when released from encapsulation within the cell wall. Additionally, the yeast cell derivatives can be more effectively utilized by targeting specific purposes.

## Proximal composition of nutritional yeast derivatives

In the context of this paper, yeast derivatives are those fractions of the yeast that derive from lysing, centrifuging, filtering and other applications done to intact yeast cells through the activities associated with downstream processing. Although there are nearly limitless types of derivatives that are possible for livestock nutrition, the principle streams are in the form of yeast cell wall and yeast cell content.

Compared to its parent stream, that being intact or nutritional yeasts, the cell wall fraction has a lower crude protein and ash content and a proportionately higher amount of carbohydrate constituents and represents between 26 and 32% of the dry weight of intact yeast cells (30). The protein in yeast cell wall tends to be in the form of glycoproteins and associated with the residual NSP and other carbohydrates present in the cell wall structure (31). However, due to incomplete processing techniques a portion of the protein in yeast cell wall originates from the cytosol of the upstream source yeast. This can be observed with the protein related standard deviation being approximately 25% of the mean and the maximum value being nearly three times that of the minimum (Table 2). Conversely, although the variability of the key carbohydrate portion of the cell wall portion has inherent variability, the relationship of standard deviation to mean value as expressed as a percentage is lower (17 vs. 25%) than that of protein. Mannan and glucan-based residues represent upwards of 95% of the non-starch polysaccharides in yeast cell wall with variability in maximal concentrations for the mannan and glucan fractions being associated with the relative degree of downstream processing, such as acid or enzyme hydrolysis, applied to any given output stream (30, 32).

**Table 2:** Nutrient composition of yeast derivatives^a^.

**Nutrient, % as is**	**Mean**	**Std Dev**	**Min**	**Max**
**Yeast cell wall (from:** ***Saccharomyces cerevisiae*****)**
Dry matter	95.81	1.62	91.91	99.71
Crude protein (*N* x 6.25)	32.53	8.13	15.81	47.22
Ether extract	0.69	1.01	n.d.^b^	5.44
Ash	3.79	1.11	2.17	5.66
Non-starch polysaccharides	37.88	6.42	27.77	54.55
Glucan/glucose	22.28	4.17	11.69	36.59
Mannan/mannose	15.49	4.68	6.74	27.30
Arabinose	0.33	0.16	0.13	0.67
Nucleotides	n.d.	n.d.	n.d.	n.d.
**Yeast extract (from:** ***Saccharomyces cerevisiae*****)**
Dry matter	96.47	0.45	95.87	97.23
Crude protein (*N* x 6.25)	53.22	11.77	43.09	69.21
Ether extract	0.28	0.18	0.03	0.65
Ash	19.12	6.36	10.35	25.79
Non-starch polysaccharides	9.32	2.25	6.77	11.05
Glucan/glucose	6.64	5.3	0.56	10.26
Mannan/mannose	2.61	3.12	0.79	6.21
Nucleotides	15.06	10.88	3.05	40.20

a From: CBS Bio-Platforms Inc. internal analyses 2020–2022.

b Not detected.

Generally speaking, the intracellular components of lysed yeast cells are referred to as yeast extracts. These extracts typically contain higher levels of both crude protein and non-protein nitrogen and lower carbohydrate concentrations than either intact yeast cells or yeast cell wall fractions (Table 2). The protein tends to be highly digestible (33), as unlike the cell wall fraction, tends to not form carbohydrate complexes, however, the presence of mannose and glucose-based polysaccharides is likely due to incomplete removal of the cell wall during downstream processing.

Containing a nitrogenous base, a ribose sugar and between one and three phosphates, nucleotides serve as the base units of the nucleic acid polymers DNA and RNA. Nucleotides are categorized by their nitrogenous base, those being guanine, adenine, cytosine and thymine in DNA, while in RNA uracil is present in place of thymine. Nucleotides play a central role in cellular metabolism, by providing energy (ATP, GTP) as well as being involved in protein synthesis and contributing to cell signaling [cGMP; (34)]. Compared to torula yeast, brewers yeast contains trace amounts of nucleotides (0.06 vs. 1.44%) with variability being high in the former as in some cases nucleotides are undetectable (Table 2). It should be noted that the variability of nucleotides in torula yeast is also high with the standard deviation being in excess of 100% of the mean as well as the maximum value being 87 times greater than the minimum (3.47 vs. 0.04%). This variability in both brewers and torula is due in part to differences of applied methods of analyses as well as factors related to production and extraction (34, 35). In addition, the means by which nucleotides are degraded from parent DNA and RNA, harvested and the conditions by which the parent cells were cultivated contribute to total and relative amounts present in any given yeast extract preparation (36).

## Targeting bioactive compounds within yeast carbohydrate fractions

The carbohydrate fraction of yeast cell wall has long been used as a source of bioactive compounds for the livestock feeding industry. Colloquially referred to as MOS (mannan-oligosaccharide), various yeast cell wall preparations have been developed that claim that the provision of MOS will increase and/or improve various aspects of health and production. However, as outlined above, mannan-based carbohydrates are present within yeast cell wall isolations along with other molecules including glucan-based polysaccharides such as beta-1,3;1,6 glucans and to a lesser extent chitin (37). Additionally, mannan exist as long chain water insoluble polysaccharides rather than an oligosaccharides (38), so the use of term MOS is a misnomer as yeast cell wall preparations contain little, if any, mannan based oligosaccharides.

A plethora of beneficial attributes have been observed and assigned to the dietary provision of yeast cell wall preparations into livestock, poultry, and aquaculture feeds. Amongst these are improvements in body weight gain and feed efficiency (39, 40) as well as augmentation of health and immunity (12, 41, 42). Other use cases have focused on the ability of yeast cell wall carbohydrates to prevent enteric infection (43) while also mitigating pathogen shedding (29) thereby bridging the gap between the classical growth and health use case into a food safety assurance technology. Looked at as a whole, it is clear that the carbohydrate portion of yeast cell wall possess bioactive properties that have the potential to beneficially augment livestock across multiple application scenarios.

As outlined above, targeting the carbohydrate fraction of yeast cells means targeting the cell wall as this is where the majority of carbohydrates reside within the yeast cell (30, 31). Within the yeast cell wall, carbohydrates exist as glycoproteins along with various minerals such as phosphorus, calcium, sodium and others. The carbohydrates are typically large polysaccharides, 10,000+ subunits in length, and composed predominantly of mannose and glucose subunits which are assembled as mannan and glucan polysaccharides. Mannose subunits within mannan polysaccharides link at locations 1,6 and 1,2- and 1,3 in alpha configurations, thereby making alpha 1,6 mannans and to a limited extent alpha 1,2 polysaccharides (44). The mannans found in yeast cell walls differ from those found in plant ingredients such as soybeans as well as palm and coconuts fruits which are also linked at the 1,6 location but are in a beta configuration and are therefore described as beta-mannans (45). Conversely, the glucose fractions are linked together at the 1,3 and 1,6 locations in beta configurations (46), thereby making beta-1,3; 1,6 glucan subunits that exist as extended beta-glucan polysaccharides. These beta-glucans differ from those found in grains such as barley, oats and rye which are connected at the 1,4 and 1,6 locations (47) but are otherwise identified as beta-glucans. The unique structure of yeast based mannans and beta-glucans impart a greater potential for biological activity compared to their plant based counterparts, the latter of which, although possessing some prebiotic properties, are generally viewed in the field of animal nutrition as an impediment to the nutritional value of their respective ingredient (37).

One commonality that all carbohydrate constituents have in common is their purported health benefits, however the degree and consistency that each inparts on the receiving animal is debatable (11, 48). To this, *Hooge* reviewed the response of broiler chickens fed yeast cell wall preparations containing both mannan and glucan components and reported that in 44 studies, an average positive increase in body weight of 1.75% was observed 79.5% of the time, while 13.6% of studies reported a decrease (49). Furthermore, when mortality was analyzed, it was reported that 40.9% of studies observed an average decrease of 16.4 while 22.7% of studies observed increases in mortality. Relatively similar results were observed for nursery pigs where a meta-analysis of 54 studies reported that an average body weight increase of 2.25% was observed in 66.6% of studies while 31.8% observed a decrease (50). Although the reported increases from these meta-analyses are promising in terms of the ability of the test preparations to improve rate of growth and reduce mortality, the fact that such high percentages reported negative responses likely relates to incomplete or incorrect targeting of the bioactive mannan and glucan fractions present within the dietary test articles under evaluation in each respective study.

Broadly speaking, the contents, or digesta, of the gastrointestinal tract can be partitioned into two sections, those being the water and water insoluble phases (51). Actions critical to digestion and enteric function, such as enzymatic breakdown of nutrients and proliferation of beneficial bacteria occur within the water-soluble phase (52). The water insoluble phase is populated by compounds such as non-emulsified fats, undigested protein complexes and various dietary fiber components (22), which although important to the overall health of the GIT, are less impactful with respect to potential benefits associated with yeast carbohydrate bioactive compounds. Thus, if a bioactive compound of carbohydrate origin is to elicit broadscale beneficial effects, it must be able to enter the water soluble phase as this is where it is able to exert the aforementioned beneficial effects. Failure to achieve entry into the water-soluble phase will limit the degree to which a bioactive compound can exert beneficial effects as it will have similar functional properties as dietary fiber (13, 22). When considering yeast carbohydrates as bioactive candidates, a critical limitation that is likely responsible for the inconsistent responses observed in the literature is that many of these preparations are highly water insoluble. For example, it was reported that a yeast cell wall preparation fed to broiler chickens that containing mostly insoluble mannans and beta-glucans had no effect on body weight gain or feed efficiency compared to the control group (53). Although the lack of improvement was not directly attributable to the structural composition of the test article, it is possible that a lack of solubility prevented a performance effect as the test article was observed to have increased intestinal villus height, which is a known end point for yeast carbohydrate bioactivity (54). The insolubility of the aforementioned test article as well as preparations in other studies that failed to show a beneficial response is related to many structural characteristics of the yeast cell wall including an extended chain length of both the mannan and glucan-based polysaccharide fractions along with the presence of associated proteins and O- and N-glycans which collectively work to reduce overall water solubility (31). Overcoming this limitation is important if functional bioactives are to be isolated and developed from the carbohydrate fraction of yeast cell wall.

It has been proposed that enzyme technology could be utilized to overcome the previously outlined solubility challenges that to date have limited the efficacy of many yeast carbohydrate bioactive materials (29, 55). There is precedence for enzyme technology application where, along with other processing technologies, carbohydrase enzymes have been successfully applied to co-products of corn starch processing resulting in the production of highly purified fructose containing oligosaccharides (FOS) possessing bioactive properties (56, 57). Similarly, exogenous enzymes have been used to generate bioactive galacooligosaccharides (GOS) from soybean and canola meal, respectively (58). With respect to yeast carbohydrates, enzymes specific to the major polysaccharides are required, namely beta 1,3-1,6 glucanase and alpha mannanase, in order to generate low molecular weight bioactive compounds *via* hydrolysis. This concept recently demonstrated the effectiveness of this approach when a purified beta 1,3-1,6 glucanase enzyme preparation was incubated along with yeast cell wall biomass in order to generate an enzymatically derived bioactive preparation composed of highly soluble, low molecular weight beta-1,3 glucan compounds along with soluble but intact mannans, that later of which have been released from the extended glucan core (29). The efficacy of this preparation was evaluated in adult laying hens where *Salmonella enteritidis* shedding within an oral challenge model was used as an endpoint and it was observed that compared to control birds as well as birds fed non-enzymatically modified yeast carbohydrates, those fed the bioactive compound had significantly reduced shedding (87 vs. 62 vs. 37%) 6 days post-inoculation as well as quantitatively reduced cecal counts of *Salmonella enteritidis* (4.30 vs. 2.95 vs. 1.90 Log10 cfu/g). When a similarly produced enzymatically produced preparation of yeast carbohydrates was fed to broiler breeder hens, it was observed that eggs laid from hens fed the bioactive material had statistically greater concentrations of IgA within the yolk (7.9 vs. 7.7 ug/ml) compared to control hens (54). Furthermore, in this same study, chicks receiving dietary supplementation of the bioactive preparation for 9 days post-hatch had longer jejunal villus heights than control chicks, but did not show any significant differences in terms of growth performance. Contrasting these largely positive outcomes on pathogen mitigation, a similar study found that supplementation of an intact mannan preparation with low water solubility, reduced quantitative Salmonella populations, non-significantly, within the ceca of 10-day old broiler chicks (27). Similar outcomes have also been observed with *E. coli* where it was demonstrated that yeast β1,3-1,6 glucans provide protection from the effect of challenge in broiler chickens (59). For both challenge models described above, it has been proposed that one potential mode of action involves yeast carbohydrates competing with pathogens that possess mannose-specific fimbriae such as *Salmonella* for specific enteric binding sites, thus decreasing attachment and colonization. In addition to this, yeast-based products can improve gut health by providing favorable conditions for intestinal *Bifidobacterium* and *Lactobacillus* spp. thus supporting their beneficial properties, including mucus production and protection of the gut integrity. Thus, the chain length and water solubility of the carbohydrate preparation being supplied directly impacts the ability of said preparation to impact pathogen control in broiler chickens.

The underlying mechanism of action of the previously described health and immunity benefits can potentially be explained at the cellular level in a study where a chicken B cell line (DT40) was incubated along with intact or enzymatically hydrolyzed yeast carbohydrates in an *in vitro* immunity evaluation model (28). Here, the authors observed that exposure to the enzymatically derived bioactive preparation compared to the unprocessed material resulted in significant increases in the expression of toll-like receptor 2b (TLR2b) and interferon gamma (INF-ɤ) within the cell line, both of which are cytokines that are involved, along with other key immunological functions (60, 61), the detection of and defense against pathogens (62, 63). Likewise, significant increases in the expression of interleukin 4 (IL-4) and 12 (IL-12) were observed in response to cellular exposure of enzymatically produced bioactive carbohydrates. This is noteworthy as both of these cytokines have roles in the management of innate immunity *via* promoting the growth and differentiation of B-cells in the case of IL-4 (64) and *via* promotion of T-cell function for IL-12, respectively (61, 65). These shifts toward a more robust innate immune response dovetail with the recently proposed concept of trained immunity and the role beta 1,3-glucans have therein (66, 67). Here, cells associated with the innate immune system recognizes beta-1,3-glucans as microbe associated molecular patterns *via* pathogen recognition receptors (66, 68). Following recognition, beta-1,3 glucans are phagocytosed and processed by innate immune systems cells present within upper intestinal lymphatic tissues, are then broken down into soluble particles and released by immune cells in order to facilitate a more robust and efficient immune response to pathogenic challenges (66, 67).

Taken together the above studies indicate that the beta-1,3 glucan and mannan fractions contained within the carbohydrate fraction of yeast cell walls have immunomodulatory properties that are in part facilitated through direct interaction with immune cell receptors that in turn promote the upregulation of cytokines that are responsible for strengthening cellular immunity. These results, combined with other similar observations (13, 27, 69), indicate the adequacy of categorizing yeast derived beta-1,3 glucan and mannans as bioactive compounds while also confirming the efficacy of said carbohydrate fractions within livestock feeds in order to promote and maintain growth performance, health and immunity.

## Bioactive targets within yeast intracellular fluid

Historically, the intracellular components of yeast have been used in the food industry as flavoring agents (70) and in the industrial microbiology and pharmaceutical industries as ingredient inputs for fermentation (71, 72). However, when used in animal nutrition the components that were of interest in other use verticals have parallel uses as bioactive targets. Chief among these are bioactive peptides (73) and nucleic acids, for which in the case of the latter, a host of research has been performed as it applies to livestock feeding programs in terms of improving growth (74) and strengthening immunity (15).

Peptides are short chains of amino acids linked by peptide bonds (75). Dipeptides, tripeptides along with chains of upto 20 amino acids are colloquially referred to as oligopeptides. From a nutritional standpoint, peptides are important as key sources of amino acids that are used in lean tissue accretion as well as in growth, development and maintenance of animals (33). However, research has shown that many peptides possess bioactive properties such as being antimicrobial (76) or being stimulative to enteric tissue development (77). However, the origin of bioactive peptides tends to be either recombinant (78) or from the hydrolysis of animal proteins such as bovine milk or fishmeal (79). Thus, given that bioactive peptides do not tend to originate in yeast cytosol, targeting this fraction of yeast for these compounds is not recommended.

As outlined previously, nucleic acids are key components of yeast extracts and typically are present as mono-, di-, or triphosphoric nucleotides (80). From a functionality point of view, nucleotides have been described as being conditionally essential (81) in that under normal metabolic conditions, *de novo* synthesis is adequate to supply biological demand. However, under conditions of stress, poor health or environmental insult, demand outstrips supply, and an exogenous dietary supply of nucleotides is required to maintain homeostasis (82). Thus, from an application standpoint, the use of yeast-derived nucleotides as bioactive compounds can become situational within a nutritional program.

To date, a number of studies have focused on best practices associated with the dietary supplication of yeast-derived nucleotides. In pigs, studies have shown that dietary supplementation of nucleotides can increase feed consumption and rate of gain of piglets (74, 83). It is thought that these improvements in performance are driven by multiple factors including improvements to intestinal morphology (15, 84) leading to increased nutrient absorption (85, 86) and improved intestinal immunity (87, 88). However, improvements tend to only be observed under unfavorable production conditions (85, 89) which narrows the applicability of yeast-based nucleotides as a dietary bioactive supplement for pigs. This narrow window whereby efficacy can be detected is likely related to the fact that the highest demand for exogenous nucleotides occurs in tissues such as the GIT which experience rapid cellular turnover immediately post-weaning and/or during infection (89, 90). Therefore, the efficacy associated with feeding yeast derived nucleotides can be optimized when provided to newly weaned piglets or preventatively to those pigs with a likelihood of encountering a stress or disease event.

Poultry species do not have the same evolved requirement for dietary nucleotides during the first weeks of life as mammals do (91). However, modern strains have been bred for rapid growth and tissue accretion and experience multiple stressor during their production cycles compared to their ancestors. It follows that due to these genetic improvements modern broiler chickens, turkeys and laying hens have an increased responsiveness to exogenous supplies of dietary yeast based nucleotides. For example, feed efficiency of broiler chickens was improved when diets were supplemented with a nucleotide containing yeast extract preparation (92, 93). In both studies it was observed that supplementation of the yeast extract led to improved GIT maturation as measured by greater ileal secretion of the digestive enzyme alkaline phosphatase as well as jejunal and ileal crypt depth. However, for contrast it was also reported that supplementing broiler chicken diets with nucleotides for 21 days had no impact on performance but led to a significant increase in ileal goblet cells (68). These results are inline with previously described observations in young pigs with the exception that in broiler chickens the benefits of nucleotide supplementation can be seen in both newly hatched and fully grown animals. Regarding impacts on health and immunity, in a study where broiler chickens were fed a nucleotide rich yeast extract and challenged with live *Eimeria* it was observed that those birds receiving the supplement had improved feed efficiency pre-challenge along with improved indices of gut function post-challenge (94). However, this same lab in a separate study observed that the provision of the same nucleotide rich yeast extract to broiler chickens had no effect on growth performance at 7 days of age but had increased bursa weight at day 35 following an *Eimeria* challenge (95).

The aquaculture industry has a long history of using yeast derived nucleotides in various feeding programs (96, 97). Originally included as a source of dietary protein (98), supplementation of yeast derived nucleotides into young fish rations has become routine practice in recent years (99). Here, the inclusion of nucleotides coincides with known periods of stress such as smoltification in salmonids (100, 101) or during early development when fish are susceptible to infection (102, 103). Compared to terrestrial animals there is strong recognition of yeast based nucleotides as bioactive compounds and adoption into commercial feeding programs is widespread.

When evaluating the efficacy of yeast-derived nucleotides purity of the preparation must always be kept in mind. This is due in most part to inconsistencies in how the extracts are manufactured which results in wide ranges in the amount and ratio of nucleotides in any given preparation. In addition, few yeast derived nucleotide preparations are manufactured solely for use in livestock nutrition, and as described above, have parallel applications in other industries. Therefore, reported results can be attributed to the presence of nucleotides within the test article but can also be linked to the presence of other yeast components such as mannans and beta 1,3-1,6-glucans which are known to be bioactive. Additionally, studies rarely declare the amount, ratio or method of analysis used to quantify each nucleotide present in a test article, which may be a contributing factor to mixed results reported to date. This inherent variability justifies the case for further identification and possible purification of yeast nucleotides in order to prove their use case as bioactives compounds.

## Future implications

Yeast derivatives have the potential to serve as sources of bioactive compounds that will have beneficial properties for livestock feeding programs. Given the accelerated movement to reduce and in most cases remove antibiotic growth promoters from livestock feeds, there is an obvious need for suitable replacement technologies. Additionally, there is a growing realization that, unlike antimicrobial compounds, such replacements are likely not to be broad-spectrum in their application strategy and that more targeted approaches will ultimately reap optimal outcomes. From this mindset one could envision multiple bioactive compounds being used to manage various challenges in any given production operation. For example, bioactive carbohydrates from yeast cell wall derivatives could be incorporated into poultry rations at certain points in the production cycle to improve body weight gain and bolster immunity and at other points to manage pathogen colonization and shedding. Additionally, nucleic acids originating in the cytosol of yeast could be incorporated into young swine and calve diets in order to support intestinal development and minimize infection related mortality and morbidity during susceptible stages of production.

## Conclusion

The challenge of understanding how best to use novel bioactive compounds will frame how this technology category incorporates itself into modern livestock production. Research that is focused on identifying and characterizing novel bioactive compounds will be required to better understand their potential. This base level knowledge capture will then require validation in multiple *in vivo* experimentation models that assess growth performance as well as broad and narrow effects on health and immunity at the cellular, tissue and systemic levels. Only once this level of comprehensive research is performed can one truly understand the beneficial potential of bioactive compounds originating from yeast derivatives.

## Data availability statement

The original contributions presented in the study are included in the article/supplementary material, further inquiries can be directed to the corresponding author.

## Author contributions

All authors listed have made a substantial, direct, and intellectual contribution to the work and approved it for publication.
